# 

**Published:** 1844-12

**Authors:** 


### CONTENTS

### OF NO. XII.

---

### ORIGINAL COMMUNICATIONS.

### ESSAYS AND CASES.

ART. I.—Cases Illustrative of the Treatment, principally with Quinine, of Inflammatory and Complicated Febrile Affections; being a Supplement to an article in the July No., for 1844, of the American Journal of Medical Sciences; "On the Treatment of Inflammatory Affections of Malarious Districts." By WM. M. BOLING, M.D., of Montgomery, Ala. - - 465

ART. II.—A Case of Rupture of the Uterus, with Artificial Anus at the point of rupture. By WILLIAM N. MORGAN, M.D., of Germantown, Tenn. - - 498

### REVIEWS.

ART. III.—An Essay towards a Correct Theory of the Nervous System. By JOHN HARRISON, M.D., Professor of Physiology and Pathology in the Medical College of Louisiana. - - - - - - - - 502

ART. IV.—An Essay on the Philosophy of Medicine. By ELISHA BARTLETT, M.D., Professor of Theory and Practice of Medicine in the University of Maryland. 506

ART. V.—The Medical Student, or Aids to the Study of Medicine. A revised and modified edition. By ROBLEY DUNGLISON, M.D., Professor of the Institutes of Medicine, &c. &c. - - - - - - - 513

ART. VI.—The Anatomy of the Human Body. By CRUVEILHIER, Professor of Anatomy to the Faculty of Medicine of Paris, &c. &c. First American, from the last Paris edition. Edited by GRANVILLE S. PATTISON, M. D., Professor of Anatomy in the University of New York; Member of the Medico-Chirurgical Society of London, &c. &c. &c. - - 518
ART. VII.—Principles of Pathology and Practice of Medicine. By JOHN MACKINTOSH, M. D.; Lecturer on the Practice of Physic, Edinburg, &c. &c. Fourth American, from last London edition. With notes and additions; by SAMUEL GEORGE MORTON, M.D., formerly Physician to the Philadelphia Hospital; author of Illustrations of Pulmonary Consumption, &c. 520
ART. VIII.—A Dictionary of Practical Medicine, comprising General Pathology, and the Nature and Treatment of Diseases, Morbid Structures, &c. By JAS. COPLAND, M.D., F.R.S. Edited, with notes; by CHARLES A. LEE, M.D. - - - - - - 521
ART. IX.—A Treatise on Pathological Anatomy. By CARL ROKITANSKY, M. D., Professor extraordinary of Pathological Anatomy of the University of Vienna.—Part I. Containing the abnormal conditions of the Organs of Respiration. Translated from the German, with additions on Diagnosis, from Shoenlein, Skoda, and others. By Dr. JOHN C. PETERS. - 522

SELECTIONS FROM AMERICAN AND FOREIGN JOURNALS.

A Case of Temporary Mental Alienation. - - - 525
Monomania Induced Through Imitation. - - - - 527
Fatal Hæmorrhage from Varix of the Vulva. - - - 530
Treatment of Chronic Catarrhal Ophthalmia. - - - 530
Observations on the Climacteric Disease, with Cases. - 531
Steel Rod Magnetised by the Influence of the will. - 533
Sciatica. - - - - - - - - - - - - 534
Deceptions in Medicine- - - - - - - - - 535
Hysterical Epilepsy. - - - - - - - - 536
Number of the Medical Profession in Great Britain. - 536

ORIGINAL INTELLIGENCE.

Traveling Letter, No. IX. - - - - - - - 537
Traveling Letter, No. X. - - - - - - - 545
Medical Classes. - - - - - - - - - 549
Yellow Fever in Woodville, Mississippi. - - - 549
Dr. Drake's Introductory Lecture. - - - - - 550
Death of Dr. Forry. - - - - - - - - - 551

